# Iberdomide in patients with systemic lupus erythematosus: a randomised, double-blind, placebo-controlled, ascending-dose, phase 2a study

**DOI:** 10.1136/lupus-2021-000581

**Published:** 2022-02-15

**Authors:** Richard A Furie, Douglas R Hough, Allison Gaudy, Ying Ye, Shimon Korish, Nikolay Delev, Michael Weiswasser, Xiaojiang Zhan, Peter H Schafer, Victoria P Werth

**Affiliations:** 1 Rheumatology, Northwell Health, Great Neck, New York, USA; 2 Clinical Research, Bristol Myers Squibb, Princeton, New Jersey, USA; 3 Translational Development, Clinical Pharmacology, Bristol Myers Squibb, Princeton, New Jersey, USA; 4 ICF Early Clinical Development, Bristol Myers Squibb, Princeton, New Jersey, USA; 5 Clinical R&D, Bristol Myers Squibb, Princeton, New Jersey, USA; 6 Biometrics, Bristol Myers Squibb, Princeton, New Jersey, USA; 7 TRC Inflammation, CV & Fibrosis and Global Health, Bristol Myers Squibb, Princeton, New Jersey, USA; 8 Corporal Michael J Crescenz VA Medical Center, University of Pennsylvania Perelman School of Medicine, Philadelphia, Pennsylvania, USA

**Keywords:** lupus erythematosus, systemic, pharmacokinetics, autoimmune diseases

## Abstract

**Objective:**

To evaluate safety, pharmacokinetics, pharmacodynamics and efficacy of iberdomide in patients with SLE. Iberdomide is a high-affinity cereblon ligand that targets the hematopoietic transcription factors Ikaros and Aiolos for proteasomal degradation.

**Methods:**

A 12-week, multicentre, double-blind, placebo-controlled, dose-escalation study in active SLE was followed by a 2-year, open-label active treatment extension phase (ATEP) (NCT02185040). In the dose-escalation phase, adults with active SLE were randomised to oral placebo or iberdomide (0.3 mg every other day, 0.3 mg once daily, 0.6 mg and 0.3 mg alternating once daily, or 0.6 mg once daily). Primary endpoints were safety and tolerability.

**Results:**

The dose-escalation phase enrolled 42 patients, with 33 completing this phase and 17 patients enrolling into the ATEP. In the dose-escalation phase, the most common treatment-emergent adverse events (TEAEs; iberdomide/placebo groups) were nausea (20.6%/12.5%), diarrhoea (17.6%/12.5%) and upper respiratory tract infection (11.8%/12.5%). Most TEAEs were mild or moderate in severity and more common in the highest dose groups in both study phases. In the dose-escalation phase, Physician’s Global Assessment and Cutaneous Lupus Erythematosus Disease Area and Severity Index (CLASI) activity scores improved relative to baseline and placebo in all iberdomide groups, with a trend toward continued score improvements in the ATEP. In the dose-escalation phase, iberdomide treatment resulted in dose-dependent reductions in total B cells and plasmacytoid dendritic cells in blood. Improvements in CLASI activity scores correlated with plasmacytoid dendritic cell depletion.

**Conclusions:**

These proof-of-concept findings suggest a favourable benefit/risk ratio in SLE for iberdomide, a drug with a novel immunomodulatory mechanism of action, supporting further clinical investigation.

**Key messages**

**What is already known about this subject?**

- SLE is a complex, heterogeneous, multisystem autoimmune disease with few approved treatments.
- Iberdomide (CC-220) is a high-affinity cereblon ligand that promotes ubiquitination and subsequent degradation of Aiolos (*IKZF3*) and Ikaros (*IKZF1*), transcription factors associated with immune cell development and SLE pathology.

**What does this study add?**

- This phase 2a study provides proof-of-concept findings suggesting a favourable benefit/risk ratio of iberdomide in patients with SLE.
- Iberdomide was well tolerated and directional clinical activity was observed for multiple endpoints.

**How might this impact on clinical practice or future developments?**

- Continued investigation of iberdomide in patients with SLE is supported by these findings.

## Introduction

SLE is a complex, heterogeneous, multisystem autoimmune disease that typically occurs in premenopausal women and can adversely affect quality of life and lead to increased morbidity and mortality.^1 2^ Despite numerous trials of novel agents, only two new agents (belimumab, anifrolumab) have been approved for the treatment of SLE in the past five decades.^2 3^ More effective and safer treatments for patients with SLE remain an unmet need, particularly in those with disease that proves refractory to conventional immunosuppressive therapy.^4^

Ikaros (*IKZF1*) and Aiolos (*IKZF3*) are transcription factors with crucial roles in immune cell development and homeostasis.^5^ *IKZF1* and *IKZF3* mRNA are overexpressed in the peripheral blood mononuclear cells of patients with SLE,^6^ contributing to lupus disease pathology.^7^ Polymorphisms in both *IKZF1* and *IKZF3* are associated with a risk of developing SLE.^8–10^

Iberdomide (CC-220) is a high-affinity cereblon ligand that promotes ubiquitination and subsequent degradation of Aiolos and Ikaros by the proteasome.^11^ In B cells from healthy donors and patients with SLE, iberdomide reduces Aiolos and Ikaros protein levels, thereby inhibiting BAFF-induced and CD40L-induced proliferation, plasmablast differentiation and IgG secretion in vitro.^7^ In cultures of peripheral blood mononuclear cells from patients with SLE, iberdomide inhibited anti-dsDNA and anti-phospholipid IgM autoantibody production in vitro.^6^

The effects of iberdomide on cell populations in healthy volunteers were examined in a single-ascending dose study.^6^ At oral doses of 0.3–6 mg once daily (QD), iberdomide decreased intracellular Aiolos levels in B cells and T cells, decreased absolute CD19+ B cell counts in peripheral blood, increased interleukin (IL)-2 and decreased IL-1β production ex vivo.^6^ In a multiple-ascending dose study in healthy volunteers, iberdomide exposure increased in a dose-proportional manner,^12^ with a terminal half-life of 9–13 hours following a single dose. Iberdomide decreased peripheral CD19+ B lymphocytes (maximum effect (E_max_), 92.4%; half maximal effective concentration (EC_50_), 0.718 ng/mL) and more modestly reduced CD3+ T lymphocytes (E_max_, 34.8%; EC_50_, 0.932 ng/mL). In ex vivo whole blood samples, levels of lipopolysaccharide-stimulated proinflammatory cytokines IL-1α and IL-1β were reduced, but anti-CD3-stimulated IL-2 and interferon (IFN)-γ levels were increased in blood from iberdomide-treated versus placebo-treated subjects. Thus, dose-dependent, differential immunomodulatory effects on B and T lymphocytes were noted in healthy volunteers receiving iberdomide, consistent with iberdomide-induced reductions in Ikaros and Aiolos levels within T lymphocytes.

The observed effects of iberdomide on B and T lymphocytes as well as cytokine levels in these studies suggest that it may represent a unique approach to the treatment of SLE. Iberdomide safety, pharmacokinetics (PK), pharmacodynamics and efficacy were therefore explored in a phase 2 proof-of-concept study of patients with SLE.

## Methods

### Trial design

CC-220-SLE-001 (NCT02185040) was a multicentre phase 2 study consisting of two parts. The 12-week, double-blind, placebo-controlled, dose-escalation phase randomised patients 4:1 (via interactive voice response system) to oral iberdomide at one of four dose levels (0.3 mg every other day (QOD), 0.3 mg QD, 0.6 mg and 0.3 mg QD on alternating days, or 0.6 mg QD), or placebo. Dose interruptions of up to 14 days were allowed for patients experiencing a clinically significant iberdomide-related adverse event (AE); for patients unable to remain on their assigned dose, iberdomide doses were reduced to the next lower dose level.

Following the 12-week dose-escalation phase, eligible patients could enrol directly into the 2-year, open-label, active treatment extension phase (ATEP). Patients may have had a period of no treatment between the study parts as they waited for study amendment to enrol in the ATEP. Those who discontinued the dose-escalation phase early or did not consent to enter the ATEP were followed for 12 weeks. The iberdomide dose in the ATEP was assigned based on the dose received in the dose-escalation phase (online supplemental figure 1).

10.1136/lupus-2021-000581.supp1 Supplementary data

### Patients

Eligible patients were ≥18 years of age with a diagnosis for ≥6 months of SLE per American College of Rheumatology 1997 criteria^13^ and a hybrid Safety of Estrogens in Systemic Lupus Erythematosus National Assessment (SELENA)-Systemic Lupus Erythematosus Disease Activity Index (SLEDAI) score^14^ of ≥4 points at baseline. This cut-off value was used to increase patient recruitment for this phase 2 study focused on safety, PK and pharmacodynamics. Patients could have qualified based on serology scores alone; however, all patients who entered the study had active disease and pertinent clinical scoring (skin and/or joints and/or other organ involvement). The hybrid SELENA-SLEDAI score is differentiated from the SELENA-SLEDAI score based on the definition of proteinuria, in which the hybrid SELENA-SLEDAI score uses >0.5 g/24 hours and the previous definition of ‘new onset or recent increase of >0.5 g/24 hours’ has been removed. Oral corticosteroids (≤10 mg prednisone or equivalent daily) and/or antimalarials (hydroxychloroquine, chloroquine, and/or quinacrine) were allowed if doses had been stable for 4 weeks prior to randomisation. No other systemic immunosuppressive treatments were permitted during the dose-escalation phase. Methotrexate, sulfasalazine or leflunomide were permitted during the ATEP. Patients may have taken alternative standard-of-care therapies between study phases. Key exclusion criteria were unstable lupus nephritis (estimated glomerular filtration rate <50 mL/min/1.73 m^2^), active central nervous system disease requiring therapeutic intervention within 6 months of screening and the presence of infectious disease.

### Endpoints and assessments

For the dose-escalation phase, the primary endpoint was safety and tolerability. The secondary endpoint was PK as measured by parameters including area under the plasma concentration-time curve calculated from time zero to infinity (AUC_inf_), maximum observed plasma concentration (C_max_), time to peak serum concentration (T_max_), terminal elimination half-life (t_1/2_), apparent clearance from plasma and apparent volume of distribution during the terminal phase. Exploratory endpoints included leucocyte subset determinations in peripheral blood on days 1, 29, 57 and 85 as measured by flow cytometry. Efficacy measures were also exploratory endpoints, and included change from baseline in Cutaneous Lupus Erythematosus Disease Area and Severity Index (CLASI) activity and damage,^15 16^ hybrid SELENA-SLEDAI, Physician’s Global Assessment (PGA) scores, and swollen and tender joint count. These were assessed on days 1, 15, 29 and 57, at final treatment visit (day 85), and during follow-up visits at 4 and 12 weeks post-treatment. In the ATEP, these efficacy measures were assessed at weeks 1, 4 and 12, every 3 months thereafter, and at final treatment and observational follow-up. In the ATEP, the primary endpoints were long-term safety and tolerability, and secondary endpoints were long-term efficacy measures.

Blood samples for PK analysis were obtained during the dose-escalation phase only. Sparse PK samples were collected from all patients predose on days 15, 29, 57 and 85. A subset of patients (four per treatment group) participated in intensive PK sampling collected on days 1 and 29 predose and at 1, 2, 3, 4, 6–8 and 24 hours postdose, in addition to sparse PK sampling times. Concentrations of iberdomide were determined by validated liquid chromatography-tandem mass spectrometry assay.^12^ Peripheral blood samples for pharmacodynamic analysis were collected at baseline, days 29 and 57, and at the final study visit.

### Statistical analysis

The safety population was defined as all patients who received ≥1 dose of iberdomide. The intent-to-treat population was defined as all patients randomised according to the protocol who received ≥1 dose of iberdomide. The PK and pharmacodynamic populations were defined as all patients in the safety population with ≥1 non-missing plasma concentration and pharmacodynamic assessment, respectively. This was a proof-of-concept study, and no formal sample size or power calculations were performed. Both parts of the trial used descriptive statistics (eg, mean, SD, and no p values were provided. AEs were coded using the Medical Dictionary for Drug Regulatory Activities V.21.0 and were summarised by system organ class, preferred term, severity and relationship to iberdomide. PK parameters were determined from patients in the intensive PK subset by non-compartmental analysis method (Phoenix WinNonLin V.6.3). For patients entering the ATEP, new baseline values for efficacy measures were determined at ATEP entry; original baseline values from dose-escalation phase were not used for ATEP efficacy analyses. Statistical analyses were performed using SAS V.9.2 or higher.

## Results

### Patient disposition and baseline characteristics

The study was initiated in December 2014 and 42 patients were enrolled in the double-blind, placebo-controlled, dose-escalation phase of the study; 93% were female, the mean age was 47 years, 64% were white (table 1). The most common concomitant medications were hydroxychloroquine (62%) and prednisone (48%; at doses ≤10 mg of prednisone equivalent). At baseline, all patients had skin and/or joint involvement as determined by CLASI and joint counts, respectively. Baseline mean (SD) CLASI scores varied considerably between treatment groups, from a low of 4.3 (5.9) in the placebo group to a high of 17.6 (12.9) in the iberdomide 0.3 mg QOD group. At baseline, 78.6% (33 of 42) and 66.7% (28 of 42) of patients had at least one tender joint or one swollen joint, respectively. Baseline mean (SD) hybrid SELENA-SLEDAI scores ranged from 5.5 (2.1) in the 0.3 mg QD group to 8.4 (4.1) in the 0.3 mg QOD group, with hybrid SELENA-SLEDAI scores in between for the other dose groups. All patients had a baseline hybrid SELENA-SLEDAI score of ≥4 except one patient who had baseline hybrid SELENA-SLEDAI score of 2 after having a qualifying hybrid SELENA-SLEDAI score at screening. In the iberdomide groups, eight patients (23.5%) discontinued prior to dose-escalation study completion, most commonly due to AEs (n=5 patients) (online supplemental figure 2; table 1).

**Table 1:** Disposition of patients and baseline* patient and disease characteristics in the dose-escalation and active treatment extension phases

n (%)	Dose escalation					ATEP	
	Placebo (n=8)	Iberdomide 0.3 mg QOD (n=8)	Iberdomide 0.3 mg QD (n=8)	Iberdomide 0.6/0.3 mg ALTN (n=9)	Iberdomide 0.6 mg QD (n=9)	Iberdomide 0.3 mg QD (n=9)	Iberdomide 0.6/0.3 mg ALTN (n=8)
Completed	7 (88)	6 (75)	7 (88)	7 (78)	6 (67)	6 (67)	1 (13)
Discontinued	1 (13)	2 (25)	1 (13)	2 (22)	3 (33)	3 (33)	7 (88)
Reasons for discontinuation							
Adverse event	1 (13)	0	0	2 (22)	3 (33)	1 (11)	4 (50)
Withdrawal by patient	0	1 (13)	0	0	0	1 (11)	1 (13)
Lack of efficacy	0	0	0	0	0	0	0
Lost to follow-up	0	1 (13)	0	0	0	0	2 (25)
Other	0	0	1 (13)	0	0	1 (11)	0
Continued to part 2	3 (38)	2 (25)	5 (63)	3 (33)	4 (44)	NA	NA
Age, mean (SD), years	44.8 (6.6)	46.0 (8.6)	48.0 (10.9)	49.8 (13.1)	47.2 (13.6)	51.2 (10.2)	47.1 (13.7)
Female, n (%)	7 (88)	8 (100)	7 (88)	8 (89)	9 (100)	8 (89)	8 (100)
Race							
White	5 (63)	6 (75)	4 (50)	7 (78)	5 (56)	4 (44)	6 (75)
Black or African American	2 (25)	2 (25)	4 (50)	1 (11)	4 (44)	5 (56)	1 (13)
Other	1 (13)	0	0	1 (11)	0	0	1 (13)
Weight, mean (SD), kg	82.0 (13.2)	78.5 (13.7)	96.3 (26.1)	74.3 (14.8)	76.5 (21.5)	87.5 (9.4)	67.5 (11.8)
Duration of SLE, mean (SD), years	13.3 (10.0)	7.9 (9.7)	9.8 (9.2)	8.2 (4.3)	10.5 (8.3)	8.9 (9.0)	12.2 (8.3)
CLASI score, mean (SD)	4.3 (5.9)	17.6 (12.9)	6.3 (9.1)	8.4 (8.6)	12.4 (16.5)	7.2 (9.0)	12.3 (10.1)
≥1 tender joint, n (%)	6 (75)	6 (75)	7 (88)	8 (89)	6 (67)	7 (78)†	3 (38)†
≥1 swollen joint, n (%)	5 (63)	5 (63)	6 (75)	8 (89)	4 (44)	6 (67)†	2 (25)†
Hybrid SELENA-SLEDAI score, mean (SD)	6.8 (1.8)	8.4 (4.1)	5.5 (2.1)	6.7 (3.2)	5.7 (1.9)	4.9 (2.9)	6.3 (2.1)
PGA score, mean (SD)	0.95 (0.52)	1.50 (0.65)	1.50 (0.61)	1.22 (0.35)	1.40 (0.60)	1.17 (0.78)	1.08 (0.62)

*For patients in ATEP previously enrolled in dose escalation, values are from time of ATEP enrolment.

†For ATEP population, number of patients with non-zero tender/swollen joint counts.

ALTN, alternating once daily; ATEP, active treatment extension phase; CLASI, Cutaneous Lupus Erythematosus Disease Area and Severity Index; NA, not applicable; PGA, Physician’s Global Assessment; QD, once daily; QOD, every other day; SELENA-SLEDAI, Safety of Estrogens in Systemic Lupus Erythematosus National Assessment-Systemic Lupus Erythematosus Disease Activity Index.

Seventeen of the 33 eligible patients (52%) enrolled in the ATEP. The median time between completion of dose escalation and the start of ATEP was 311 days (range, 3–527) and the last study visit occurred in September 2018. Nine patients received iberdomide 0.3 mg QD; eight received iberdomide 0.6/0.3 mg on alternating days. Demographics and characteristics of patients entering the ATEP were similar to those in the dose-escalation phase. Ten (59%) patients in the ATEP discontinued treatment, including five (29%) who discontinued due to treatment-emergent AEs (TEAEs), of whom four (24%) were in the iberdomide 0.6/0.3 mg on alternating days and one (6%) was in the iberdomide 0.3 mg QD group.

### Safety

Mean (SD) treatment duration in the double-blind, placebo-controlled, dose-escalation phase ranged from 9.5 (4.4) to 11.8 (0.5) weeks (online supplemental table 1). TEAEs were reported in five (62.5%) patients in the placebo group and 30 (88.2%) patients in the iberdomide groups (table 2). Among iberdomide-treated patients in the dose-escalation phase, the most common TEAEs were nausea (20.6%), diarrhoea (17.6%) and upper respiratory tract infection (11.8%) (table 2). All four patients with vitamin D deficiency had low vitamin D levels prior to receiving iberdomide. Neutropenia and pneumonia (8.8% each) were reported in the two highest iberdomide dose groups.

**Table 2:** Overview of treatment-emergent adverse events (TEAEs)

n (%)	Dose escalation					ATEP	
	Placebo (n=8)	Iberdomide 0.3 mg QOD (n=8)	Iberdomide 0.3 mg QD (n=8)	Iberdomide 0.6/0.3 mg ALTN (n=9)	Iberdomide 0.6 mg QD (n=9)	Iberdomide 0.3 mg QD (n=9)	Iberdomide 0.6/0.3 mg ALTN (n=8)
Any TEAE	5 (62.5)	7 (87.5)	7 (87.5)	8 (88.9)	8 (88.9)	9 (100)	7 (87.5)
Any treatment-related TEAE	1 (12.5)	2 (25.0)	2 (25.0)	4 (44.4)	6 (66.7)	2 (22.2)	5 (62.5)
Severe TEAE	1 (12.5)	0	0	1 (11.1)	2 (22.2)	0	5 (62.5)
Serious TEAE	2 (25.0)	0	0	1 (11.1)	1 (11.1)	0	4 (50.0)
Any serious treatment-related TEAE	0	0	0	0	1 (11.1)	0	0
TEAEs leading to drug withdrawal	1 (12.5)*	0	0	2 (22.2)†	3 (33.3)‡	1 (11.1)§	4 (50.0)¶
TEAEs leading to death	0	0	0	0	0	0	0
TEAEs in >5% of patients**							
URTI	1 (12.5)	1 (12.5)	1 (12.5)	1 (11.1)	1 (11.1)	4 (44.4)	3 (37.5)
Diarrhoea	1 (12.5)	1 (12.5)	1 (12.5)	2 (22.2)	2 (22.2)	1 (11.1)	3 (37.5)
Nausea	1 (12.5)	1 (12.5)	3 (37.5)	3 (33.3)	0	2 (22.2)	0
Bronchitis	0	0	2 (25.0)	0	0	3 (33.3)	3 (37.5)
Urinary tract infection	0	1 (12.5)	0	1 (11.1)	0	2 (22.2)	2 (25.0)
Neutropenia	0	0	0	1 (11.1)	2 (22.2)	1 (11.1)	1 (12.5)
Pneumonia	0	0	0	2 (22.2)	1 (11.1)	1 (11.1)	1 (12.5)
Cough	0	0	0	1 (11.1)	1 (11.1)	0	2 (25.0)
Nasopharyngitis	1 (12.5)	0	0	0	1 (11.1)	2 (22.2)	0
Pain in extremity	0	1 (12.5)	0	0	1 (11.1)	2 (22.2)	0
Vitamin D deficiency††	0	0	1 (12.5)	2 (22.2)	1 (11.1)	0	0
Vomiting	1 (12.5)	0	1 (12.5)	1 (11.1)	1 (11.1)	0	0
Maculopapular rash	0	1 (12.5)	0	1 (11.1)	1 (11.1)	0	0
Musculoskeletal pain	0	0	0	0	0	1 (11.1)	2 (25.0)
Osteoarthritis	0	0	0	1 (11.1)	0	2 (22.2)	0
Sinusitis	0	0	0	0	0	2 (22.2)	1 (12.5)

*Deep vein thrombosis.

†Pneumonia/diarrhoea and ecchymosis/maculopapular rash.

‡Pneumonia, dermatitis and neutropenia.

§Small fibre neuropathy.

¶Bronchitis, gastroenteritis, decreased neutrophil count, oral candidiasis, and pulmonary embolism.

**In combined dose-escalation and ATEP safety populations (N=59).

††Present prior to treatment.

ALTN, alternating once daily; ATEP, active treatment extension phase; QD, once daily; QOD, every other day; URTI, upper respiratory tract infection.

In the longer duration ATEP (mean (SD) duration 75.6 (32.9) weeks (iberdomide 0.3 mg QD) and 49.5 (37.4) weeks (iberdomide 0.6/0.3 mg on alternating days)), TEAEs were also primarily gastrointestinal or infectious in origin. The most common were upper respiratory tract infection (41.2%), bronchitis (35.3%), diarrhoea (23.5%) and urinary tract infection (23.5%). In both the dose-escalation phase and the ATEP, most TEAEs were of mild or moderate severity.

During the dose-escalation phase, one patient each receiving iberdomide 0.6/0.3 mg on alternating days and iberdomide 0.6 mg QD group had severe pneumonia, and one patient in the latter group had severe neutropenia. During the ATEP, five severe TEAEs (gastroenteritis, seizure, vitreous detachment, pulmonary embolism and SLE) were reported in the iberdomide 0.6/0.3 mg alternating days group, whereas no patient in the iberdomide 0.3 mg QD group had a severe TEAE.

During the dose-escalation phase, serious TEAEs were reported in two iberdomide-treated patients (5.9%; both pneumonia in the two highest iberdomide dose groups, one of which was considered treatment related) and in two (25.0%) patients in the placebo group (deep vein thrombosis, schizoaffective disorder and SLE). In the ATEP, serious TEAEs occurred in four patients (23.5%; pulmonary embolism, seizure, SLE and vitreous detachment); all were observed with the higher iberdomide dose (0.6/0.3 mg on alternating days), and none were considered treatment related.

In both the dose-escalation phase and the ATEP, as expected, patients receiving iberdomide who had lower neutrophil counts at baseline were more likely to experience a higher grade of neutropenia. The neutropenia was reversible, and patients recovered to prior neutrophil counts after temporary interruption of iberdomide. No other clinically meaningful changes in laboratory values, vital signs, weight or ECG findings were observed during the dose-escalation phase or the ATEP.

Eight iberdomide-treated patients (23.5%) discontinued prior to completion of the dose-escalation phase, most commonly due to AEs (five patients (14.7%)). These consisted of pneumonia (n=2), neutropenia, dermatitis, diarrhoea, ecchymosis and maculopapular rash (n=1 each); none were in the iberdomide 0.3 mg dose groups. In the ATEP, the higher iberdomide dose group (0.6/0.3 mg on alternating days) had a higher incidence of TEAEs leading to treatment interruption or withdrawal (four of eight (50%); bronchitis, gastroenteritis, decreased neutrophil count, oral candidiasis and pulmonary embolism, one each) than the iberdomide 0.3 mg QD group (one of nine (11.1%); small fibre neuropathy). No opportunistic infections were reported and no clinically meaningful changes in vital signs or ECG findings were observed. Two cases of thromboembolic events were observed in the study: one pulmonary embolism in the iberdomide 0.3/0.6 alternating days group in the ATEP and one deep vein thrombosis in the placebo group during the double-blind treatment period; both subjects discontinued the study with no known complications. There were no fatal AEs in the study.

### Efficacy

**Figure 1:**
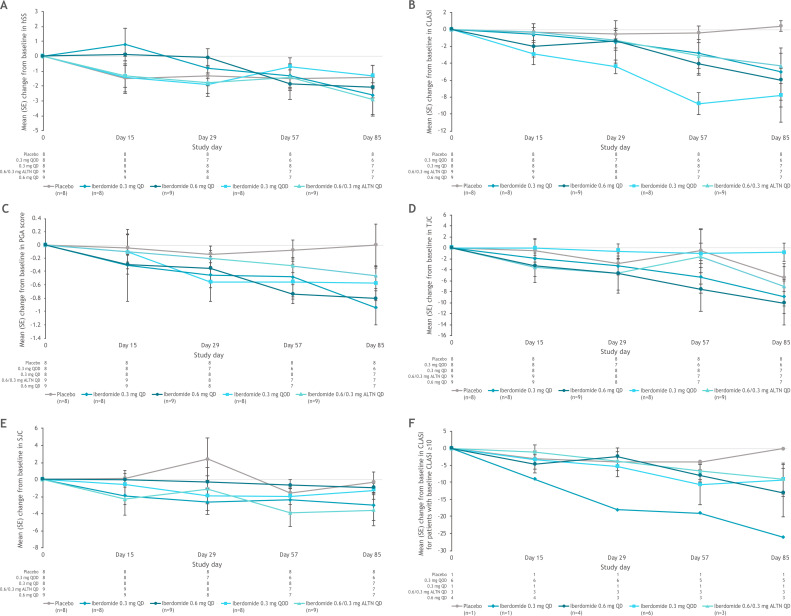
Mean change from baseline in (A) hybrid SELENA-SLEDAI scores, (B) CLASI scores, (C) PGA scores, D) tender joint count (TJC) and (E) swollen joint count (SJC) in the ITT population during the dose escalation part, and (F) mean change from baseline in CLASI score in patients with baseline CLASI ≥10. ALTN, alternating once daily; CLASI, Cutaneous Lupus Erythematosus Disease Area and Severity Index; hSS, hybrid SELENA-SLEDAI; ITT, intent to treat; PGA, Physician’s Global Assessment; QD, once daily; QOD, every other day; SELENA-SLEDAI, Safety of Estrogens in Systemic Lupus Erythematosus National Assessment-Systemic Lupus Erythematosus Disease Activity Index.

Efficacy was an exploratory objective in this study, which was not powered for statistical significance; however, evidence of clinical activity with iberdomide was observed for multiple endpoints. In the placebo-controlled dose-escalation phase, mean hybrid SELENA-SLEDAI scores showed a trend toward improvement in all groups, including placebo (figure 1A). At day 85, 22%–50% of patients in the iberdomide groups achieved a ≥4-point reduction from baseline in hybrid SELENA-SLEDAI scores^17^ compared with 13% in the placebo group. Improvements were greatest in the iberdomide 0.6/0.3 mg alternating days group, with a mean (SD) reduction of 2.9 (3.4) points on day 85. The trend toward hybrid SELENA-SLEDAI score improvement continued during the ATEP in both iberdomide groups, with improvement in the 0.3 mg QD group consistently greater than in the 0.6/0.3 mg alternating days group after week 4 (online supplemental figure 3). At week 96, two of five patients in the 0.3 mg QD group and none in the 0.6/0.3 mg alternating days group had achieved a ≥4-point reduction in hybrid SELENA-SLEDAI score from ATEP baseline.

In the dose-escalation phase, a trend toward improvement from baseline in CLASI score through week 12 was observed in the iberdomide groups, with minimal changes observed in the placebo group (figure 1B). At day 85, the mean (SD; percentage change from baseline) change from baseline in CLASI scores was +0.4 (1.8; +9.5%) in the placebo group and −7.8 (10.2; −35.3%), −5.0 (9.6; −63.4%), −4.3 (6.3; −25.3%), and −6.0 (9.7; −39.8%) in the iberdomide 0.3 mg QOD, 0.3 mg QD, 0.6/0.3 mg alternating days, and 0.6 mg QD groups, respectively. The minimal clinically important difference in CLASI score of −4 (16) was exceeded in all iberdomide groups but not in the placebo group at day 85, and a trend toward improved CLASI score over time in the iberdomide groups was also evident among patients with baseline CLASI score ≥10 (figure 1F). Improvements in CLASI score relative to baseline also were observed in the ATEP by the first post-baseline time point (week 1), and mean values remained below ATEP baseline values throughout the treatment period (online supplemental figure 4).

In the dose-escalation phase, PGA scores improved in all iberdomide treatment groups (but not the placebo group), exceeding the minimal clinically important difference of −0.3^1^ ^18^ at days 57 and 85 (figure 1C). The greatest improvements in PGA scores at day 85 were noted in the iberdomide 0.3 mg QD and 0.6 mg QD groups. Improvements in PGA scores from ATEP baseline through week 96 were also noted in the ATEP (online supplemental figure 5). At ATEP week 96, mean (SD; percentage change from baseline) PGA scores were −0.52 (0.6; −47.8%) and −0.20 (no SD (n=1); −14.3%) relative to ATEP baseline in the iberdomide 0.3 mg QD and 0.6/0.3 mg alternating days groups, respectively.

In the dose-escalation phase, tender joint counts and swollen joint counts appeared to decrease in all iberdomide dose groups at day 85 (figure 1D and E). This trend continued during the ATEP in the iberdomide 0.3 mg group only.

### PK and pharmacodynamics

PK samples were available for 33 patients; all were included in the PK analysis. Iberdomide plasma PK was characterised by rapid absorption, with C_max_ occurring at a median T_max_ of 2–6 hours for all dose levels (online supplemental table 2). The geometric mean t_1/2_ ranged from approximately 8–12 hours. Plasma concentrations showed dose-related increases between dosing groups (online supplemental figure 6), with accumulation ratios of approximately 1.6 (AUC and C_max_) upon multiple dosing in non-alternating cohorts at steady state. A PK/pharmacodynamic analysis demonstrated decreasing CD19 B cells and plasmacytoid dendritic cells (pDCs) with increasing exposure to iberdomide (online supplemental figure 7A, B). There was no relationship between iberdomide plasma levels and CD3 T cells or neutrophils at the doses studied, but an increase in peripheral blood plasma cells with increasing iberdomide C_trough_ levels was observed (online supplemental figure 7C–E).

**Figure 2:**
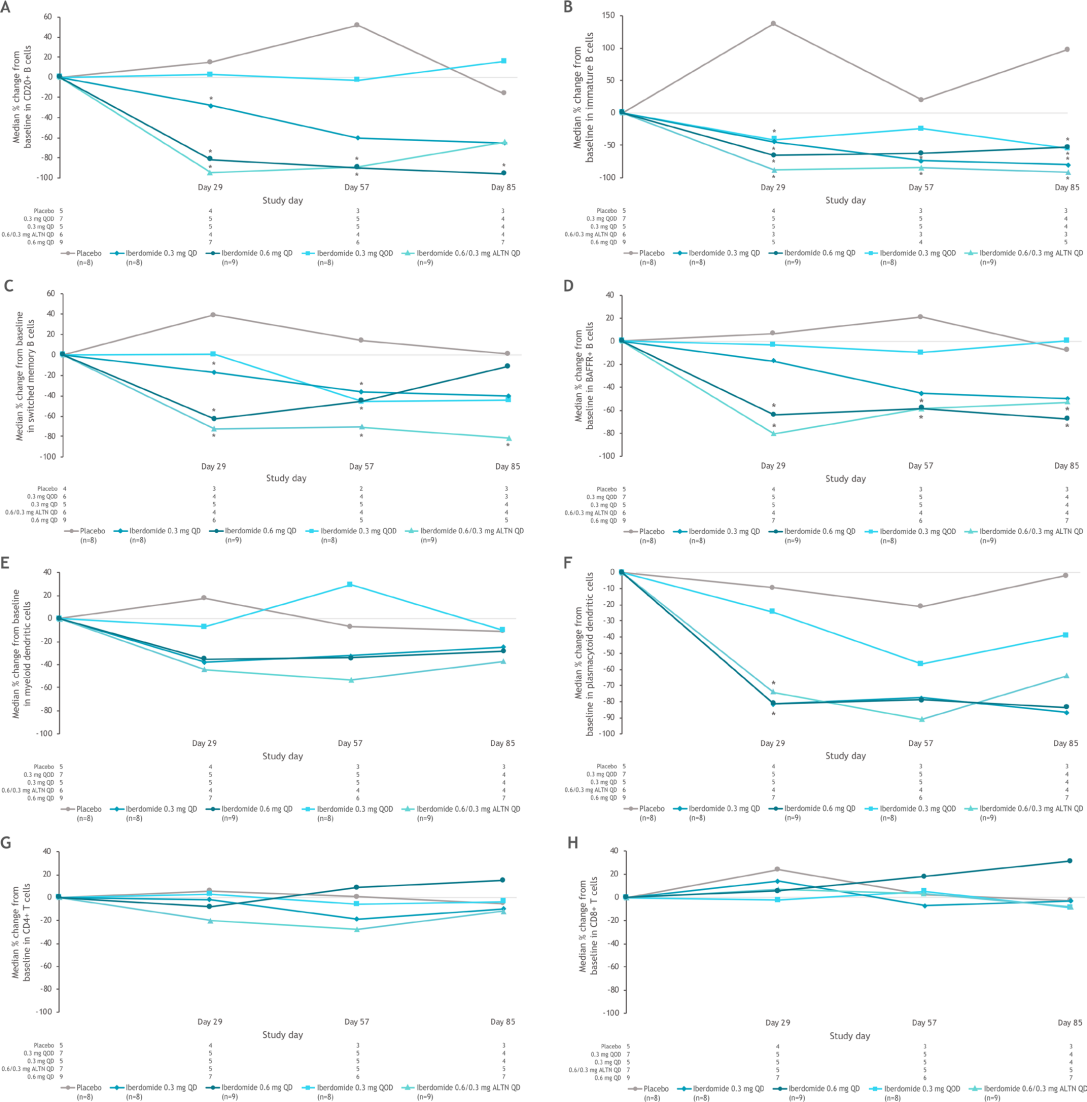
Changes in (A) CD20+ B cells, (B) immature B cells, (C) switched memory B cells, (D) BAFFR+ B cells, (E) myeloid dendritic cells, (F) plasmacytoid dendritic cells, (G) CD4+ T cells, (H) CD8+ T cells following dosing with placebo or iberdomide in the part 1 PD population. *Significantly different from placebo using two-sided 95% CIs based on analysis of covariance model with the percentage change from baseline as response variable and the treatment and baseline score as factors. ALTN, alternating once daily; BAFFR+, B-cell-activating factor receptor positive; QD, once daily; QOD, every other day.

**Figure 3:**
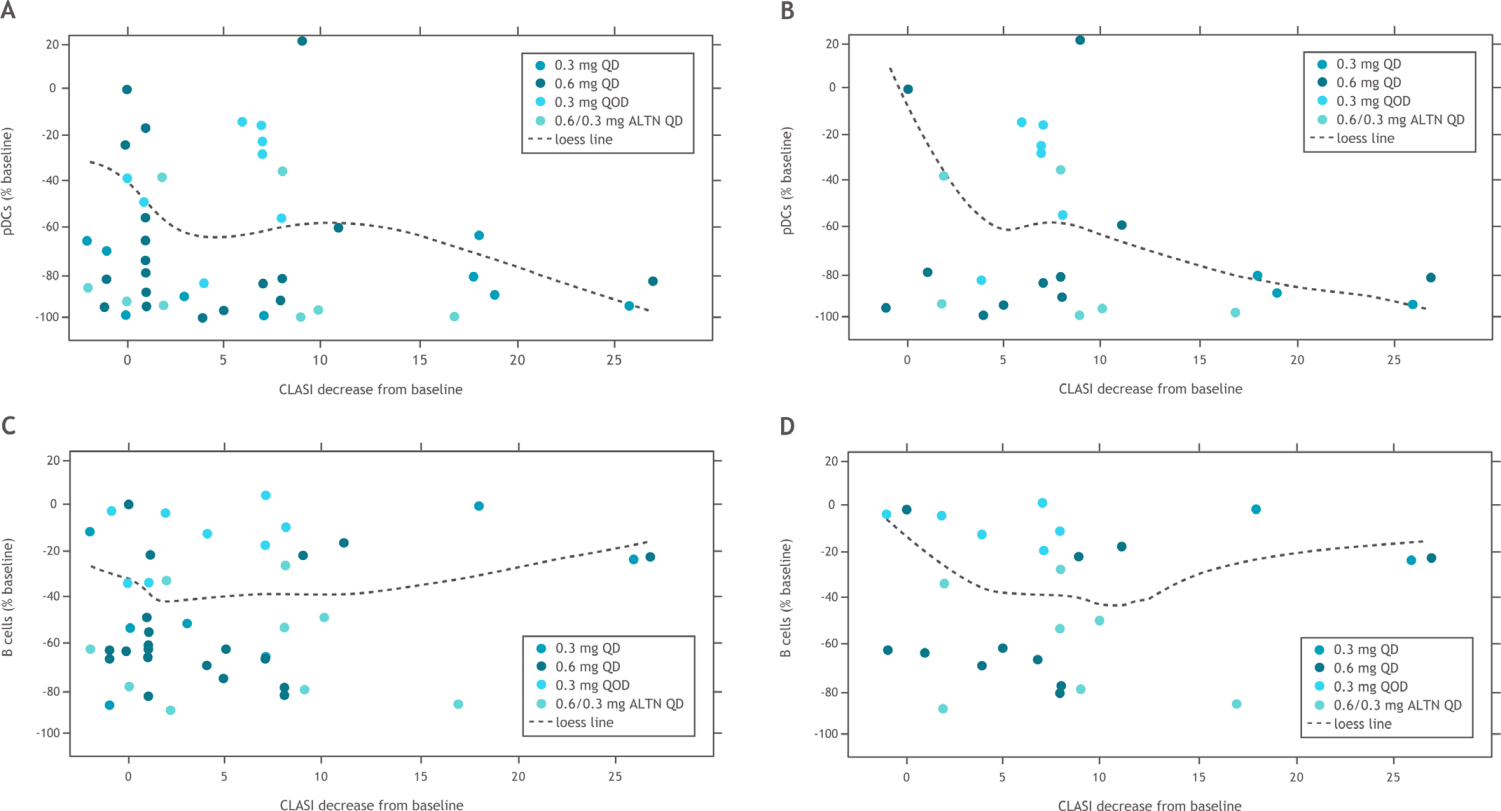
Relationships between change in CLASI score and (A) pDCs in blood in all iberdomide-treated patients, (B) pDCs in blood in iberdomide-treated patients with baseline CLASI score ≥10, (C) B cells in blood in all iberdomide-treated patients and (D) B cells in blood in iberdomide-treated patients with baseline CLASI score ≥10. ALTN, alternating QD; CLASI, Cutaneous Lupus Erythematosus Disease Area and Severity Index; pDCs, plasmacytoid dendritic cells; QD, once daily; QOD, every other day.

Iberdomide treatment resulted in dose-dependent reductions in total CD20 B cells, immature B cells, switched memory B cells and CD268 (BAFFR) B cells as well as pDCs (figure 2). There were no significant changes in CD4+ T cells in any groups; at the highest iberdomide dose group, 0.6 mg QD, there was a trend for an increase in CD8+ T cells (figure 2). Anti-dsDNA antibody levels were within the normal range at baseline for many patients (with median values of 12 IU/mL in each treatment group). The least-squares mean increase in anti-dsDNA was 99.6 IU/mL in the placebo group at week 12, and the iberdomide groups had moderate mean increases or decreases (<25 IU/mL) over 12 weeks. There was a strong correlation between CLASI improvement and pDC depletion in all patients (figure 3A), as well as in patients with baseline CLASI score ≥10 (figure 3B). In contrast, depletion of B cells did not correlate with improvements in CLASI score in either of these groups (figure 3C, D).

## Discussion

Iberdomide, an orally available agent which binds to the cereblon E3 ubiquitin ligase complex, results in the proteasomal degradation of key immune cell transcriptional regulators Ikaros and Aiolos. Preclinical and ex vivo evidence suggests that iberdomide could represent a unique approach to the treatment of SLE. The present two-part proof-of-concept study was designed primarily to determine the safety and tolerability of iberdomide in patients with SLE.

In both the dose-escalation phase and the ATEP, the most commonly reported TEAEs in patients receiving iberdomide were gastrointestinal events and infections; most were mild or moderate in severity. TEAE frequency and severity appeared to be dose dependent. Serious or severe TEAEs were observed only in the highest iberdomide dose groups (0.6/0.3 mg alternating days and 0.6 mg QD); most discontinuations due to TEAEs occurred in the two highest iberdomide dose groups.

Neutropenia was an expected AE. In vitro studies indicated an inhibitory effect of iberdomide on neutrophil maturation, but with full recovery of normal maturation following a 5-day drug-free period (data on file). In the present study, neutropenia AEs were relatively infrequent (11.1% and 12.5% in the iberdomide 0.3 mg QD and 0.6/0.3 mg alternating days groups, respectively) over the prolonged course of treatment (median 95.9 and 60.6 weeks, respectively) in the ATEP. Cases of neutropenia were addressed with temporary dose interruptions with recovery to the prior count level in most instances. The long duration of iberdomide treatment suggests that iberdomide is well tolerated and TEAEs can be effectively managed.

Iberdomide was rapidly absorbed, with dose-related increases in systemic exposure over the dose range. Efficacy, as previously noted, was an exploratory endpoint in this study, which was not powered for hypothesis testing and for which statistical significance was not assessed. Mean PGA scores improved relative to baseline in all iberdomide groups (but not the placebo group) during dose escalation and continued to improve over the course of the ATEP, which was not placebo controlled. Hybrid SELENA-SLEDAI scores trended toward improved values in all groups, including placebo in the dose-escalation phase of the study, but with a greater percentage of patients achieving reductions of ≥4 points relative to baseline among iberdomide groups. Results for tender and swollen joint counts in both study parts were mixed during dose escalation, with most iberdomide groups tending toward reduced mean values over time. These efficacy measures in the ATEP showed similar trends through 96 weeks (final ATEP visit).

During the dose-escalation phase, a trend toward numerically improved CLASI scores through week 12 was observed for all iberdomide treatment groups (but not the placebo group) relative to baseline values. Notably, although the lowest dose iberdomide group (0.3 mg QOD) appeared to have the greatest reductions in CLASI scores, patients in this cohort also had the highest mean CLASI score of any group at baseline (17.6). Baseline values in the other treatment groups ranged from 4.3 to 12.4. These baseline differences and the small sample size limit conclusions regarding dose effects of iberdomide. When analysing the pharmacodynamic immune cell data, improvements in CLASI score were correlated with observed depletion in pDCs (the principal source of IFN-α^19^) but not B cells, among all iberdomide-treated patients as well as in those with baseline CLASI score ≥10. These findings suggest that in patients with SLE treated with iberdomide, the trend toward improvement in the cutaneous manifestations of SLE was related to pDC depletion but not B cell number and/or activity. Although the IFN signature was not examined in the current study, these observations are consistent with iberdomide-mediated reduction in type I IFN response as its operative mechanism of action.

The PK/pharmacodynamic analysis revealed a relationship between iberdomide drug exposure levels and decreases in pDCs and B cells, as well as an unexpected increase in peripheral blood plasma cells. The increased plasma cell numbers in the blood, observed mainly in the 0.6 mg QD dose group, might be driven by an increased production of IL-2 and IL-10, which can act as plasma cell differentiation factors.^20^ This observed increase in plasma cells, combined with the upward trend in CD8+ T cells, suggests that the 0.6 mg QD iberdomide dose may result in undesirable pharmacodynamic effects in the context of lupus treatment. No dose-dependent effects of iberdomide were observed on anti-dsDNA antibody levels at 12 weeks; however, a subsequent study showed a dose-dependent decrease in these antibody levels starting after 16 weeks of treatment in patients with baseline levels ≥8 IU/mL.^21^

Study limitations include the small sample size, the sizeable time gap between the dose-escalation phase and the ATEP, and the limited (12 weeks) duration of the dose-escalation phase, a particularly short period given that 4 weeks may be required for the full pharmacodynamic effects of iberdomide to manifest. A patient population with mild disease activity enrolled in this study based on the relatively low mean baseline hybrid SELENA-SLEDAI scores (4.9–8.4) and the exclusion of patients receiving >10 mg/day of prednisone or any background immunosuppressants. Lower CLASI and PGA scores in the placebo group at baseline limit the ability to identify treatment differences for these outcomes.

Our findings suggest a tolerable short-term (dose escalation) and long-term (ATEP) safety profile for iberdomide in patients with SLE, consistent with that reported in the first-in-human study.^12^ Multiple validated measures, particularly PGA and CLASI scores, provided directional evidence of iberdomide efficacy. Taken as a whole, findings from this proof-of-concept study support further investigation of iberdomide in patients with SLE.

## Data Availability

BMS policy on data sharing may be found at https://www.bms.com/researchers-and-partners/independent-research/data-sharing-request-process.html.
