# Supplementary material for: Iberdomide in patients with systemic lupus erythematosus: a randomised, double-blind, placebo-controlled, ascending-dose, phase 2a study
Source: Lupus Sci Med. 2022 Feb 15;9(1):e000581. doi: 10.1136/lupus-2021-000581 (PMC8852715; doi:10.1136/lupus-2021-000581)
Supplement: Supplementary data [file lupus-2021-000581supp001.pdf]

SUPPLEMENTAL APPENDIX

A Randomized, Double-Blind, Placebo-Controlled, Ascending-Dose, Phase 2a Study of Iberdomide in Patients With Systemic Lupus Erythematosus

Richard A. Furie[1], Douglas R. Hough[2*], Allison Gaudy[2], Ying Ye[2], Shimon Korish[2], Nikolay Delev[2], Michael Weiswasser[2], Xiaojiang Zhan[2], Peter H. Schafer[2], Victoria P. Werth[3]

[1]Northwell Health, Great Neck, New York, USA; [2]Bristol Myers Squibb, Princeton, New Jersey, USA; [3]Corporal Michael J Crescenz VA Medical Center and University of Pennsylvania Perelman School of Medicine, Philadelphia, Pennsylvania, USA

* At the time of the study.

Corresponding Author:

Richard Furie, MD

Division of Rheumatology

Northwell Health

865 Northern Boulevard, Suite 302

Great Neck, NY 11021

516 708 2550

rfurie@northwell.edu

ORCID: 0000-0001-6712-1585

Supplemental Table 1. Mean treatment duration in the dose escalation and active treatment extension phase.

	Dose Escalation					ATEP	
Weeks, (SD)	Placebo (n=8)	Iberdomide 0.3 mg QOD (n=8)	Iberdomide 0.3 mg QD (n=8)	Iberdomide 0.6/0.3 mg QD (n=9)	Iberdomide 0.6 mg QD (n=9)	Iberdomide 0.3 mg QD (n=9)	Iberdomide 0.3/0.6 mg QD (n=8)
Mean treatment duration	11.8 (0.5)	10.2 (3.5)	11.5 (1.5)	10.2 (3.5)	9.5 (4.4)	75.6 (32.9)	49.5 (37.4)

QD, once daily; QOD, every second day; SD, standard deviation.

Supplemental Table 2. Geometric mean (geometric CV%) iberdomide plasma PK parameters by dose group after dosing on Day 29 of Part 1.

Dose Group	N	C_{max} (ng/mL)	AUC_t (ng⋅hr/mL)	T_{max}[a] (h)	$t_{1/2}$ (h)
0.3 mg QOD	3	1.02 (4.3)	13.34 (14.1)	4.00 (2.1-4.1)	8.46[b] (NA)
0.3 mg QD	3	1.09 (1.8)	15.55 (1.8)	2.00 (2.0-3.05)	11.85 (4.1)
0.6/0.3 mg ALTN[c]	3	2.37 (42.7)	24.85 (110.5)	3.00 (1.0-4.0)	9.39[d] (11.1)
0.6 mg QD	3	3.51 (51.7)	52.65 (82.4)	2.02 (1.1-3.1)	11.32[d] (4.8)

ALTN, alternating once-daily; AUC_t, area under the concentration-time curve calculated from time zero to the last measured time point (over a 24-hour period); C_{max}, maximum observed plasma concentration; CV%, coefficient of variation; NA, not applicable; PK, pharmacokinetics; QD, once daily; QOD, every other day; $t_{1/2}$, terminal elimination half-life; T_{max}, time to maximum plasma concentration.

[a]Median (minimum-maximum).

[b]n = 1.

[c]Day 29 dose was 0.6 mg.

[d]n = 2.

Furie RA, *et al. Lupus Sci Med* 2022; 9:e000581. doi: 10.1136/lupus-2021-000581

Supplemental Figure 1. Selection of iberdomide doses in the ATEP.

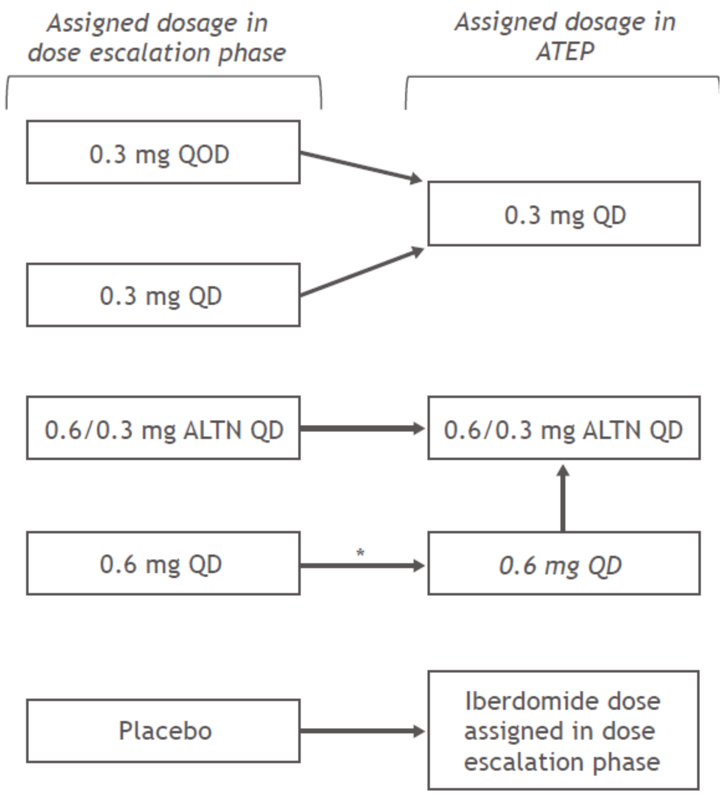

*Patients in the iberdomide 0.6 mg QD group in the dose-escalation phase were initially assigned to the same dose in the ATEP, but later switched to 0.6/0.3 mg ALTN after the 0.6 mg QD dose was removed in a protocol amendment.

ALTN, alternating once-daily dose; ATEP, active treatment extension phase; QD, once daily; QOD, every other day.

Furie RA, *et al. Lupus Sci Med* 2022; 9:e000581. doi: 10.1136/lupus-2021-000581

Supplemental Figure 2. Patient disposition in the dose escalation and active treatment extension phases.

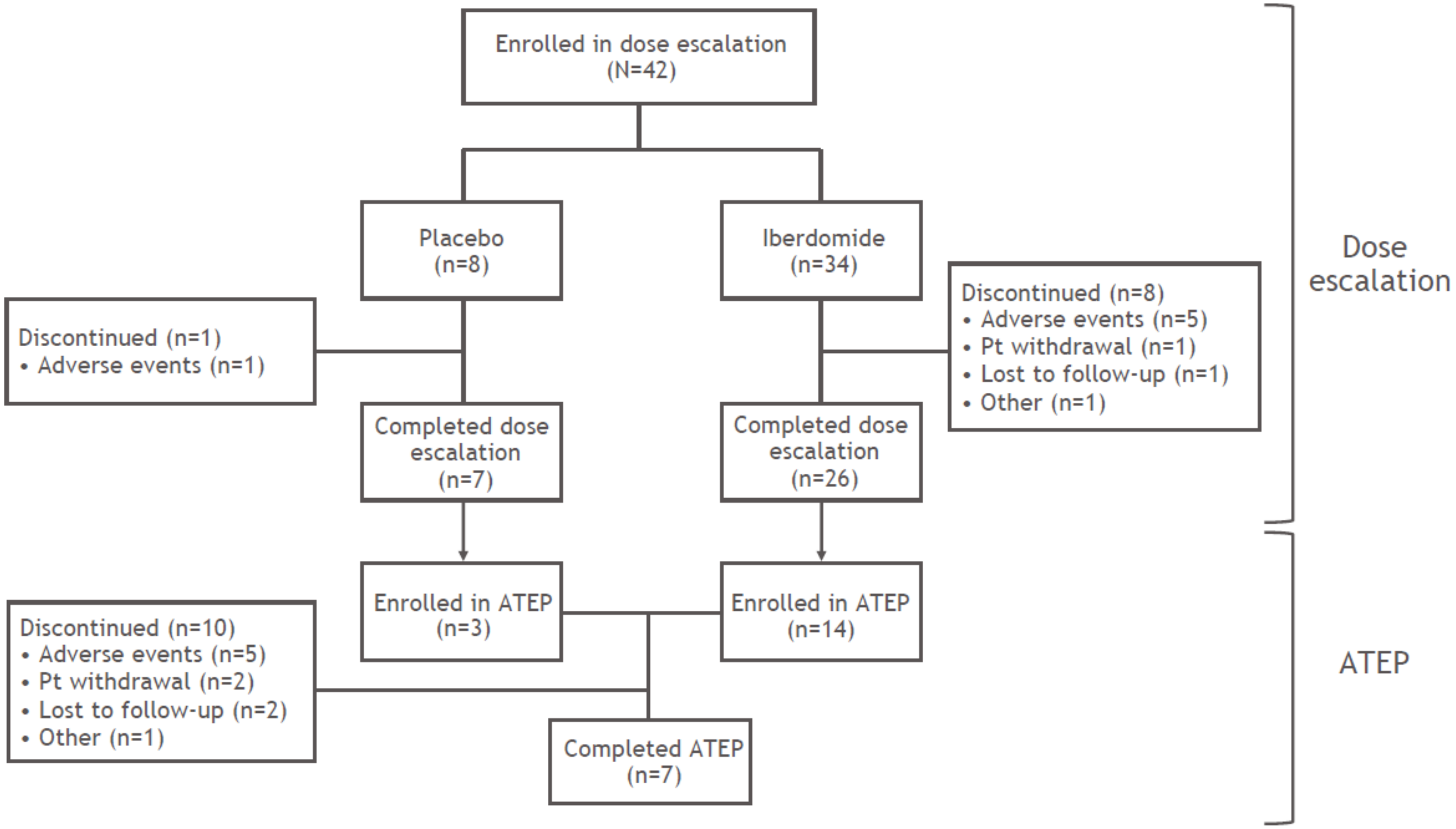

ATEP, active treatment extension phase; pt, patient.

Supplemental Figure 3. Mean (SE) change in hybrid SELENA-SLEDAI score from baseline by time point in the ATEP population.

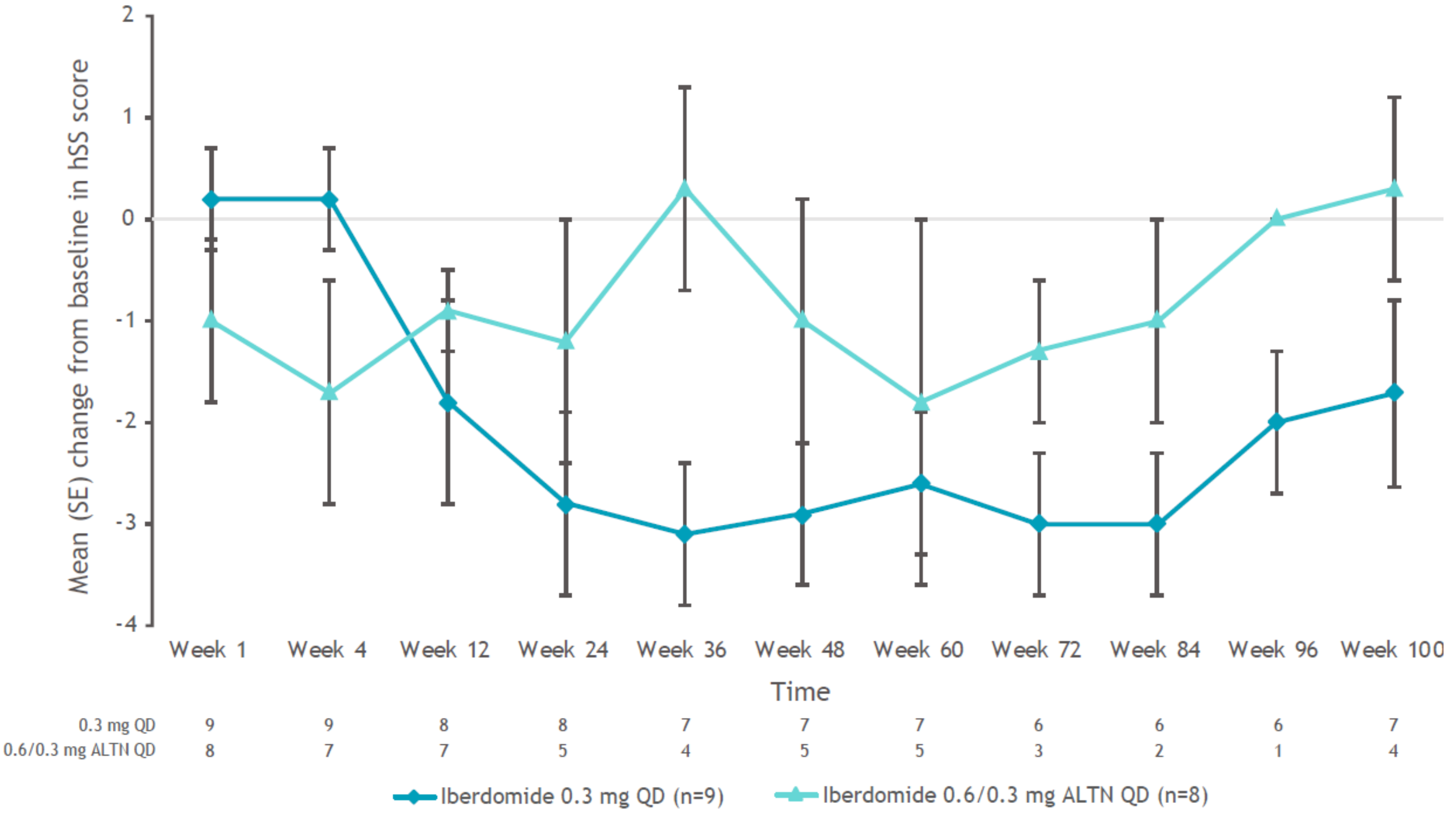

ATEP, active treatment extension phase; QD, once daily; SELENA, Safety of Estrogens in Systemic Lupus Erythematosus National Assessment; SE, standard error of mean; SLEDAI, Systemic Lupus Erythematosus Disease Activity Index.

Furie RA, *et al. Lupus Sci Med* 2022; 9:e000581. doi: 10.1136/lupus-2021-000581

Supplemental Figure 4. Mean (SE) change in CLASI activity score from baseline by time point in the ATEP population.

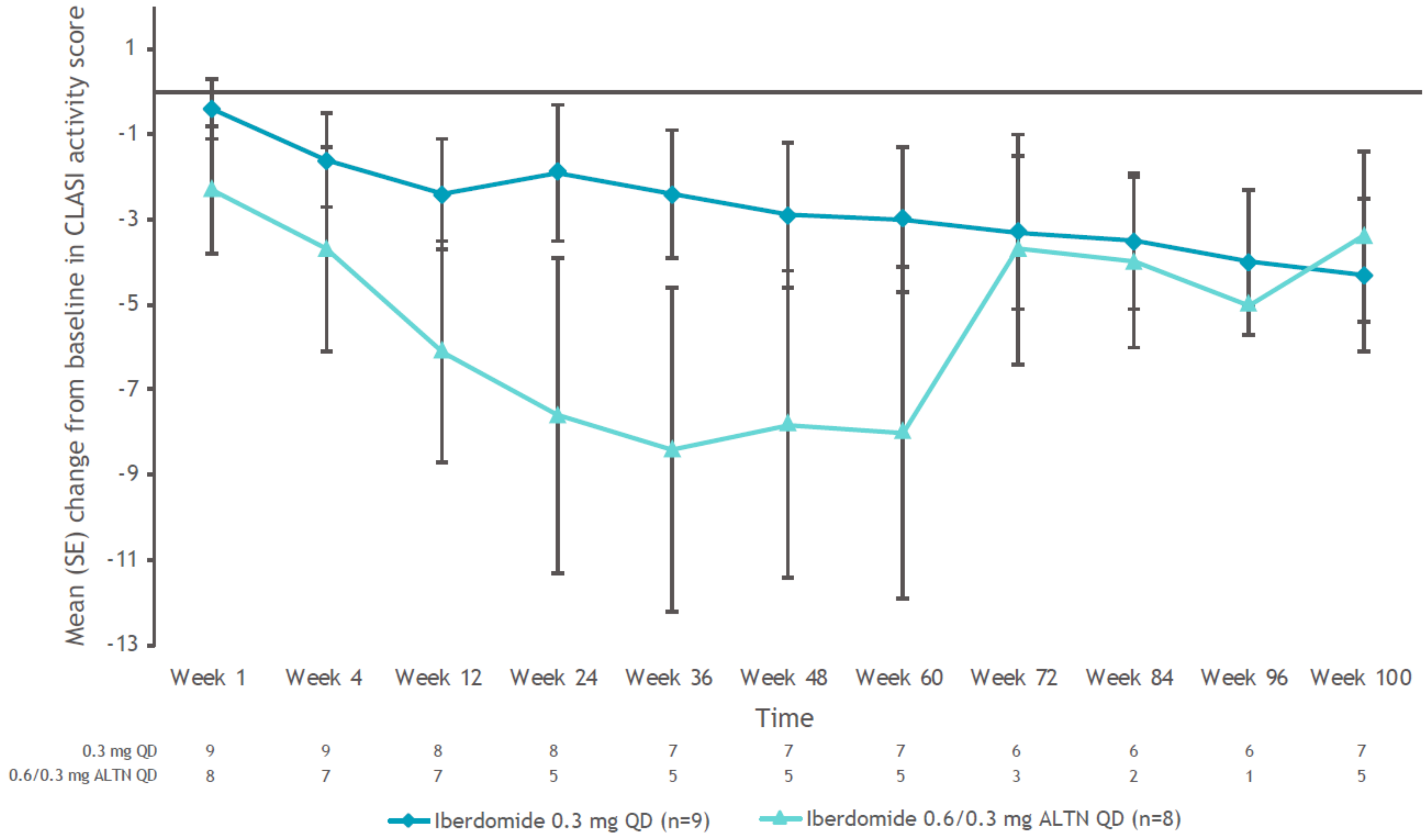

CLASI, Cutaneous Lupus Area and Severity Index; QD, once daily; SE, standard error of the mean.

Supplemental Figure 5. Mean (SE) change in PGA score from baseline by time point in the ATEP population.

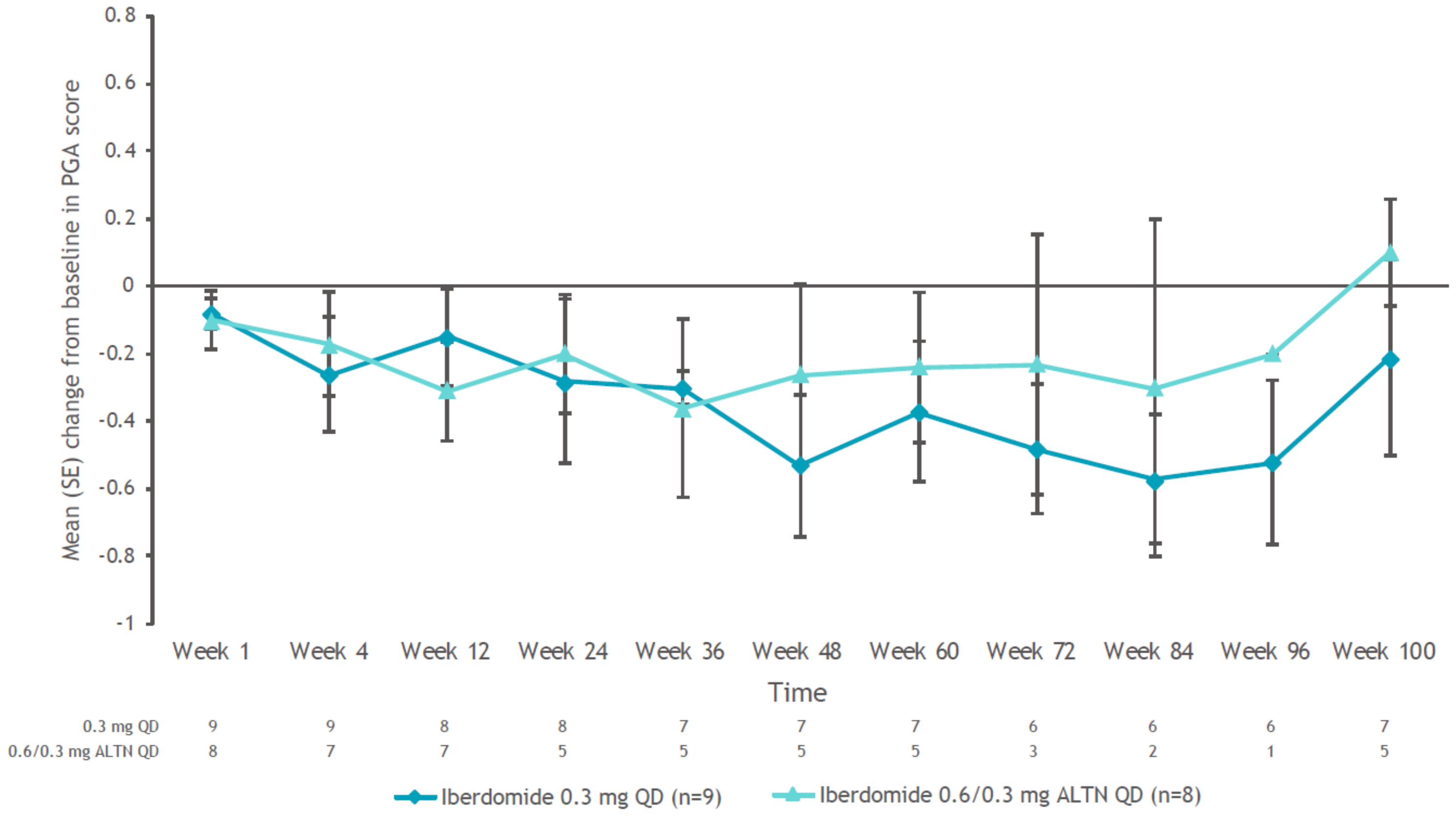

ATEP, active treatment extension phase; PGA, Physicians Global Assessment; QD, once daily; SE, standard error of mean.

Supplemental Figure 6. Mean iberdomide plasma concentration by dose on Day 29. Error bars indicate standard deviation.

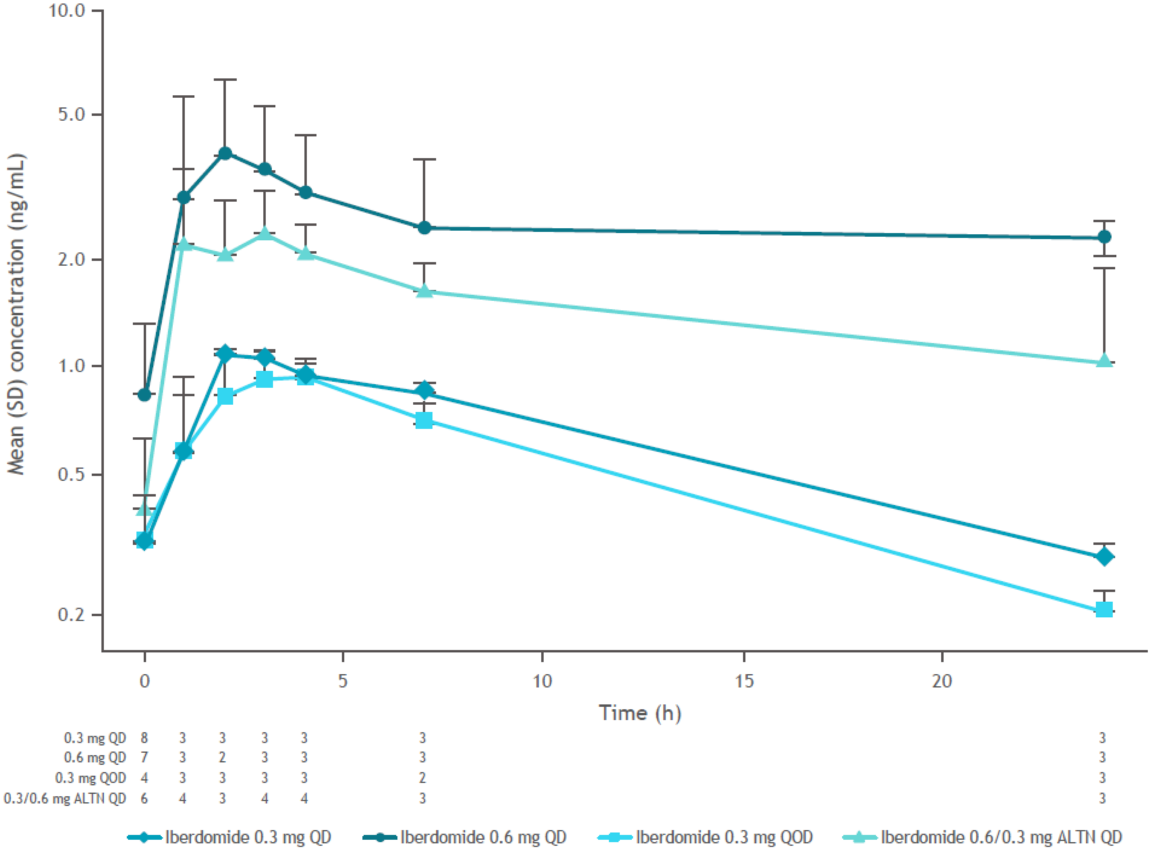

Supplemental Figure 7. Relationship between iberdomide plasma concentrations and peripheral blood (**A**) CD19 B cells, (**B**) plasmacytoid dendritic cells, (**C**) CD3 T cells, (**D**) neutrophils, and (**E**) plasma cells in PK/PD evaluable patients in the dose escalation phase.

A

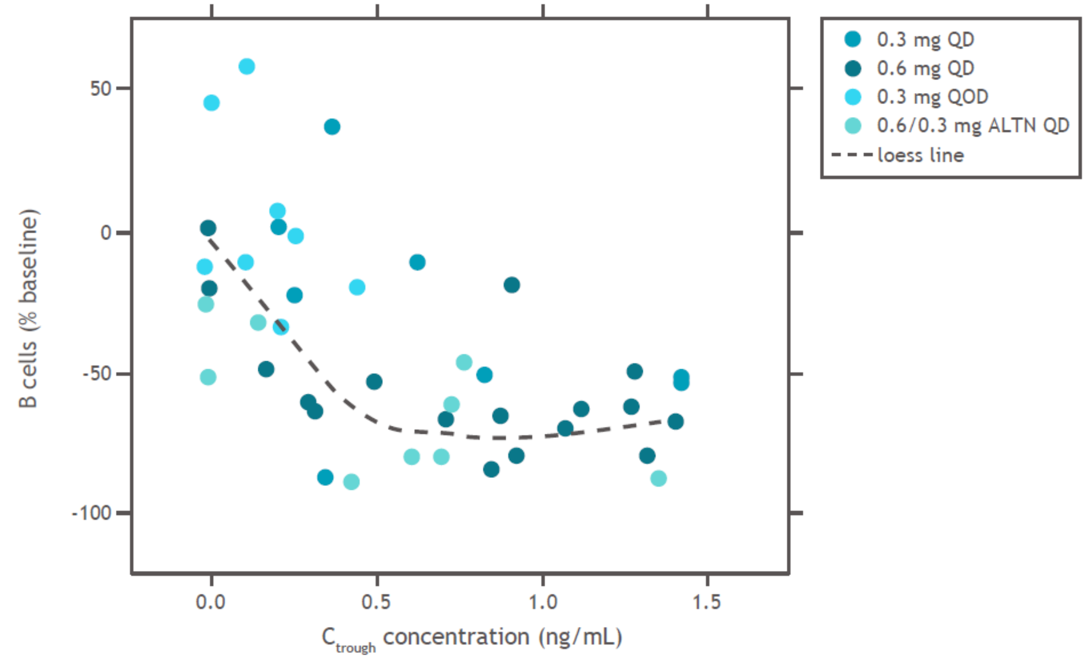

Furie RA, *et al. Lupus Sci Med* 2022; 9:e000581. doi: 10.1136/lupus-2021-000581

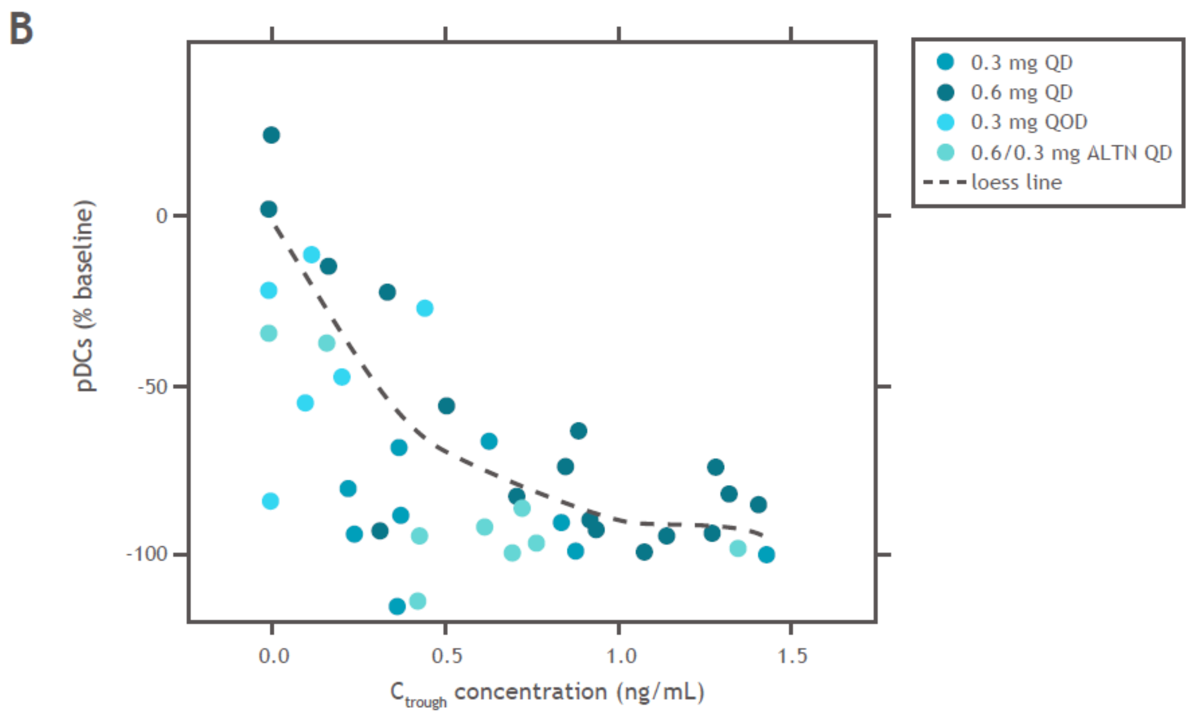
B
0
-50
-100
0.0
0.5
1.0
1.5
pDCs (% baseline)
C_{trough} concentration (ng/mL)
0.3 mg QD
0.6 mg QD
0.3 mg QOD
0.6/0.3 mg ALTN QD
loess line

C

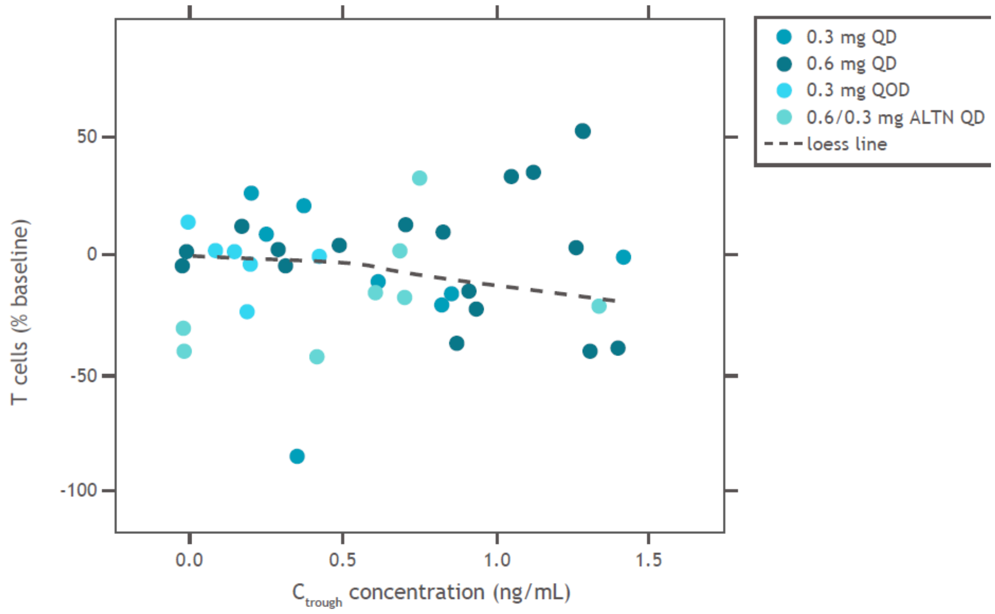

Furie RA, *et al. Lupus Sci Med* 2022; 9:e000581. doi: 10.1136/lupus-2021-000581

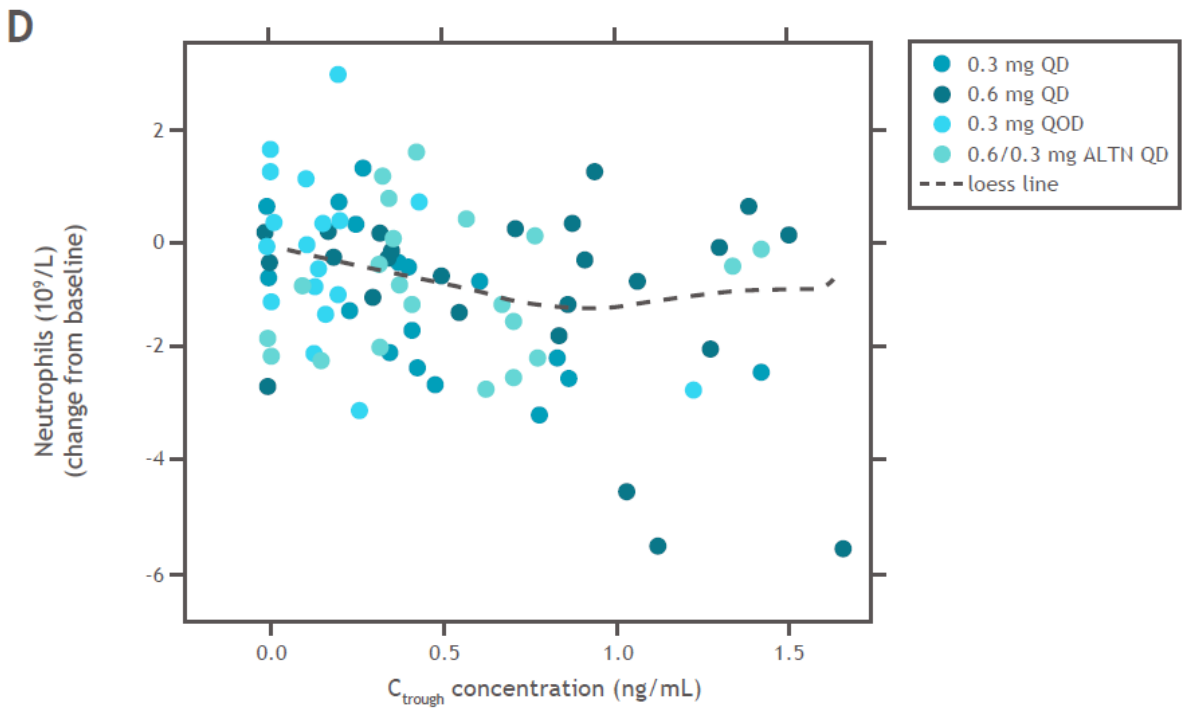
D
Neutrophils (10⁹/L)
(change from baseline)
2
0
-2
-4
-6
0.0
0.5
1.0
1.5
C trough concentration (ng/mL)
0.3 mg QD
0.6 mg QD
0.3 mg QOD
0.6/0.3 mg ALTN QD
loess line

13

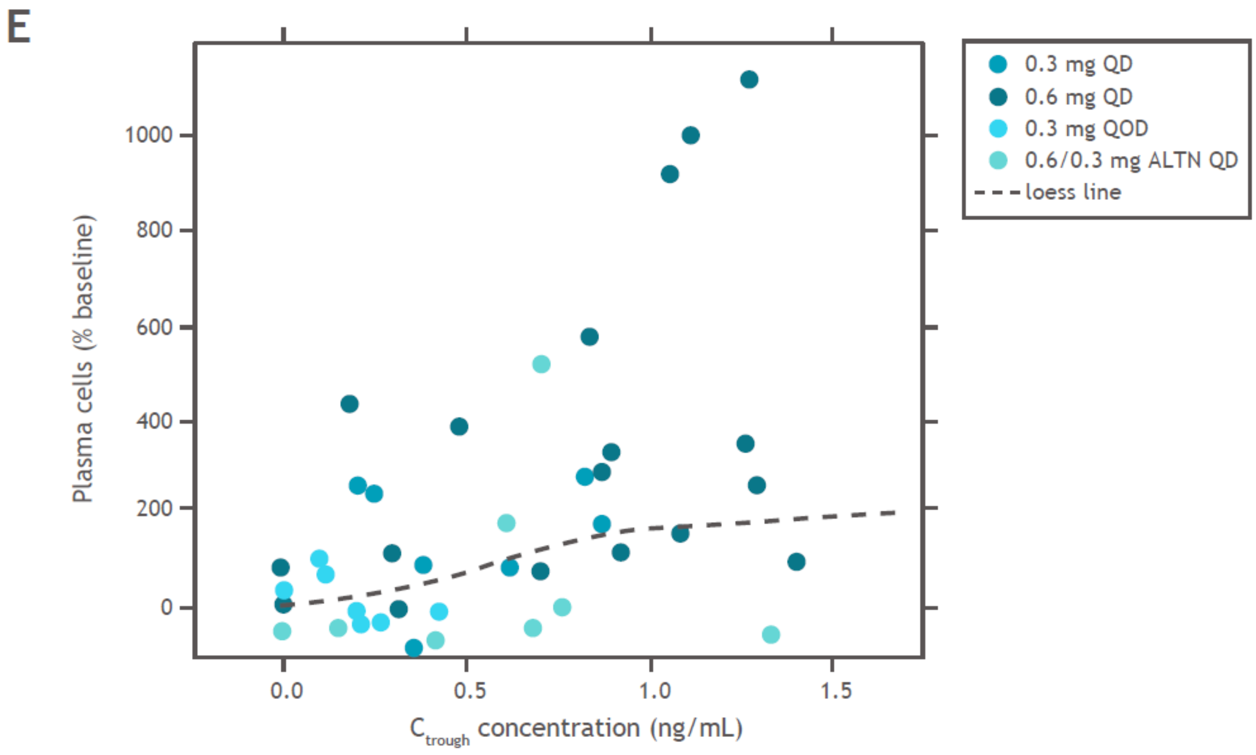

C_{trough}, minimum or trough concentration observed after drug administration and just prior to administration of subsequent dose; QD, once daily; QOD, every other day.
